# Medical News

**Published:** 1855-05

**Authors:** 


### MEDICAL NEWS.

### AMERICAN MEDICAL ASSOCIATION.

The Association will commence its session in the Musical Fund Hall, Locust above Eighth, on Tuesday, May 1st, at 11 A. M.

The Committee on Registration will attend at the Hall of the College of Physicians, in Spruce above 8th, on Saturday, April 28th, between 11 and 2 o'clock in the morning, and at 4 o'clock and 7 in the afternoon, and on Monday, from 11 till 2, P. M., and from 4 till 10, P. M., and at the Musical Fund Hall, at 8 A. M. on Tuesday, to register city delegates and any others who may present themselves. It is to be hoped that this early opportunity will be taken advantage of by the city delegation, as much delay and trouble will be thereby avoided.

Arrangements by which delegates will pass to and from Philadelphia at half price, on presentation of their credentials, have been made with the following Railroad Companies: Pennsylvania Railroad from Pittsburgh to Philadelphia; Baltimore and Philadelphia Railroad, from Baltimore to Philadelphia; Baltimore and Ohio Railroad, from Wheeling to Baltimore; Petersburg and Roanoke Railroad, in Virginia; and the chain of roads from Elmira to Philadelphia.

---

A table of Urinary Deposits, with their Microscopical and Chemical Tests, has been sent us. It is a complete map, as it were, of urinary affections, which must have given its author, Dr. John King, of Cincinnati, much trouble to put together. We regard it as a very useful paper, particularly to the young student.

---

Dr. Horace Green has retired from the Chair of Practice in the New York Medical College. Dr. Henry G. Cox has been elected his successor.

---

MEDICAL SOCIETY OF THE STATE OF PENNSYLVANIA.—The Annual Session of the Society will be held in Hollidaysburg on the last Wednesday (30th) of May, at 10 o'clock in the forenoon.

The Secretaries of the several County Societies will please forward the lists of delegates to either of the Secretaries.

D. FRANCIS CONDIE, Philadelphia,
HENRY CARPENTER, Lancaster,
*Secretaries.*

---

NOTE.—Our attention having been directed to p. 205 of our last number, where it is stated that a sponge "dropped into the windpipe of a man whose larynx Dr. Peaslee was cauterizing," we take pleasure in correcting a slight but most unintentional error contained in those words. The statement should have been that *the man himself* "dropped a sponge into his windpipe, and had tracheotomy performed for its extraction. The poor man died of hemorrhage into the trachea." (Watson, p. 19.) The operators were Drs. Crosby and Peaslee.

